# Genomic Epidemiology of an Endoscope-Associated Outbreak of *Klebsiella pneumoniae* Carbapenemase (KPC)-Producing *K*. *pneumoniae*

**DOI:** 10.1371/journal.pone.0144310

**Published:** 2015-12-04

**Authors:** Jane W. Marsh, Mary G. Krauland, Jemma S. Nelson, Jessica L. Schlackman, Anthony M. Brooks, A. William Pasculle, Kathleen A. Shutt, Yohei Doi, Ashley M. Querry, Carlene A. Muto, Lee H. Harrison

**Affiliations:** 1 Infectious Diseases Epidemiology Research Unit, University of Pittsburgh School of Medicine and Graduate School of Public Health, Pittsburgh, Pennsylvania, United States of America; 2 Public Health Dynamics Laboratory, University of Pittsburgh Graduate School of Public Health, Pittsburgh, Pennsylvania, United States of America; 3 Division of Microbiology, University of Pittsburgh Medical Center, Pittsburgh, Pennsylvania, United States of America; 4 Division of Infectious Diseases, University of Pittsburgh School of Medicine, Pittsburgh, Pennsylvania, United States of America; 5 Division of Hospital Epidemiology and Infection Control, University of Pittsburgh Medical Center, Pittsburgh, Pennsylvania, United States of America; Sidra Medical and Research Center, QATAR

## Abstract

Increased incidence of infections due to *Klebsiella pneumoniae* carbapenemase (KPC)-producing *Klebsiella pneumoniae* (KPC-Kp) was noted among patients undergoing endoscopic retrograde cholangiopancreatography (ERCP) at a single hospital. An epidemiologic investigation identified KPC-Kp and non-KPC-producing, extended-spectrum β-lactamase (ESBL)-producing Kp in cultures from 2 endoscopes. Genotyping was performed on patient and endoscope isolates to characterize the microbial genomics of the outbreak. Genetic similarity of 51 Kp isolates from 37 patients and 3 endoscopes was assessed by pulsed-field gel electrophoresis (PFGE) and multi-locus sequence typing (MLST). Five patient and 2 endoscope isolates underwent whole genome sequencing (WGS). Two KPC-encoding plasmids were characterized by single molecule, real-time sequencing. Plasmid diversity was assessed by endonuclease digestion. Genomic and epidemiologic data were used in conjunction to investigate the outbreak source. Two clusters of Kp patient isolates were genetically related to endoscope isolates by PFGE. A subset of patient isolates were collected post-ERCP, suggesting ERCP endoscopes as a possible source. A phylogeny of 7 Kp genomes from patient and endoscope isolates supported ERCP as a potential source of transmission. Differences in gene content defined 5 ST258 subclades and identified 2 of the subclades as outbreak-associated. A novel KPC-encoding plasmid, pKp28 helped define and track one endoscope-associated ST258 subclade. WGS demonstrated high genetic relatedness of patient and ERCP endoscope isolates suggesting ERCP-associated transmission of ST258 KPC-Kp. Gene and plasmid content discriminated the outbreak from endemic ST258 populations and assisted with the molecular epidemiologic investigation of an extended KPC-Kp outbreak.

## Introduction

Infections caused by carbapenemase-producing *Klebsiella pneumoniae* have recently emerged as a serious public health problem. A recent surveillance study from the United States demonstrated that 6.1% of *K*. *pneumoniae* had resistance to either imipenem or meropenem [1]. Resistance to carbapenems is often conferred by production of plasmid-encoded *K*. *pneumoniae* carbapenemase (KPC). KPC confers resistance to most β-lactam antibiotics leaving few treatment options for serious infections [2]. Moreover, the location of the KPC gene on a functional transposon on self-transmissible plasmids has resulted in widespread dissemination of carbapenem resistance to a variety of Gram-negative pathogens [3,4]. A molecular epidemiologic investigation of KPC-producing *K*. *pneumoniae* (KPC-Kp) collected from 1996 to 2008 identified sequence type 258 (ST258) as a dominant lineage in the United States and established substantial KPC plasmid diversity [5]. Recent reports demonstrate that ST258 KPC-Kp has spread globally and is the cause of numerous hospital-associated outbreaks [6–8]. The mechanism of ST258 KPC-Kp persistence is unclear. However, comparative whole genome sequence analyses have established ST258 as a hybrid clone that recently emerged through multiple recombination events [9,10]. These studies suggest that genetic recombination at specific regions of divergence or genetic “hotspots” may contribute to ST258 clonal success [9,10]. Whole genome and plasmid sequence analysis have demonstrated utility for outbreak detection and transmission tracking of ST258 [6,11–13].

KPC incidence is routinely tracked at our facility. During 2012, UPMC Presbyterian Hospital (UPMC), a 762-bed teaching hospital affiliated with the University of Pittsburgh, experienced an increase in the incidence of KPC-Kp. Epidemiologic data were collected and analyzed. Gastrointestinal (GI) procedures including endoscopic retrograde cholangiopancreatography (ERCP) were identified as potential sources of KPC-Kp transmission. A potential index case dating back to April 2011 was identified suggesting that an ERCP endoscope may have become contaminated with a KPC-Kp isolate from this patient and that high-level disinfection had failed, thus permitting transmission through subsequent ERCP procedures. To investigate this possibility, all GI endoscopes were cultured post routine high-level disinfection. One ERCP endoscope (Scope A) was found to be contaminated with extended-spectrum β-lactamase (ESBL)-producing Kp (ESBL-Kp) and KPC-Kp and one endoscopic ultrasound (EUS) scope (Scope B) was positive for ESBL-Kp as previously reported [14,15]. A second ERCP endoscope (Scope C) contaminated with KPC-Kp post high-level disinfection demonstrated the potential of ERCP-mediated KPC-Kp transmission. Genotyping by PFGE was performed on KPC-Kp and ESBL-Kp isolates from patients with Scope A exposure, as well as a convenience sample of isolates collected from patients with or without history of any GI endoscopy and isolates obtained from endoscopes A, B and C. Whole genome sequence (WGS) was performed on a subset of patient and endoscope isolates. In addition, plasmid profiling and sequencing of 2 distinct KPC-Kp plasmids was performed to investigate the source of the KPC gene and plasmid diversity among the ST258 outbreak isolates. The overall objective of this study was to characterize the genotypes of patient and endoscope KPC-Kp isolates associated with the outbreak and to examine the microbial genomic properties of ST258 at our institution.

## Materials and Methods

### Isolates and patients

**Fig 1:**
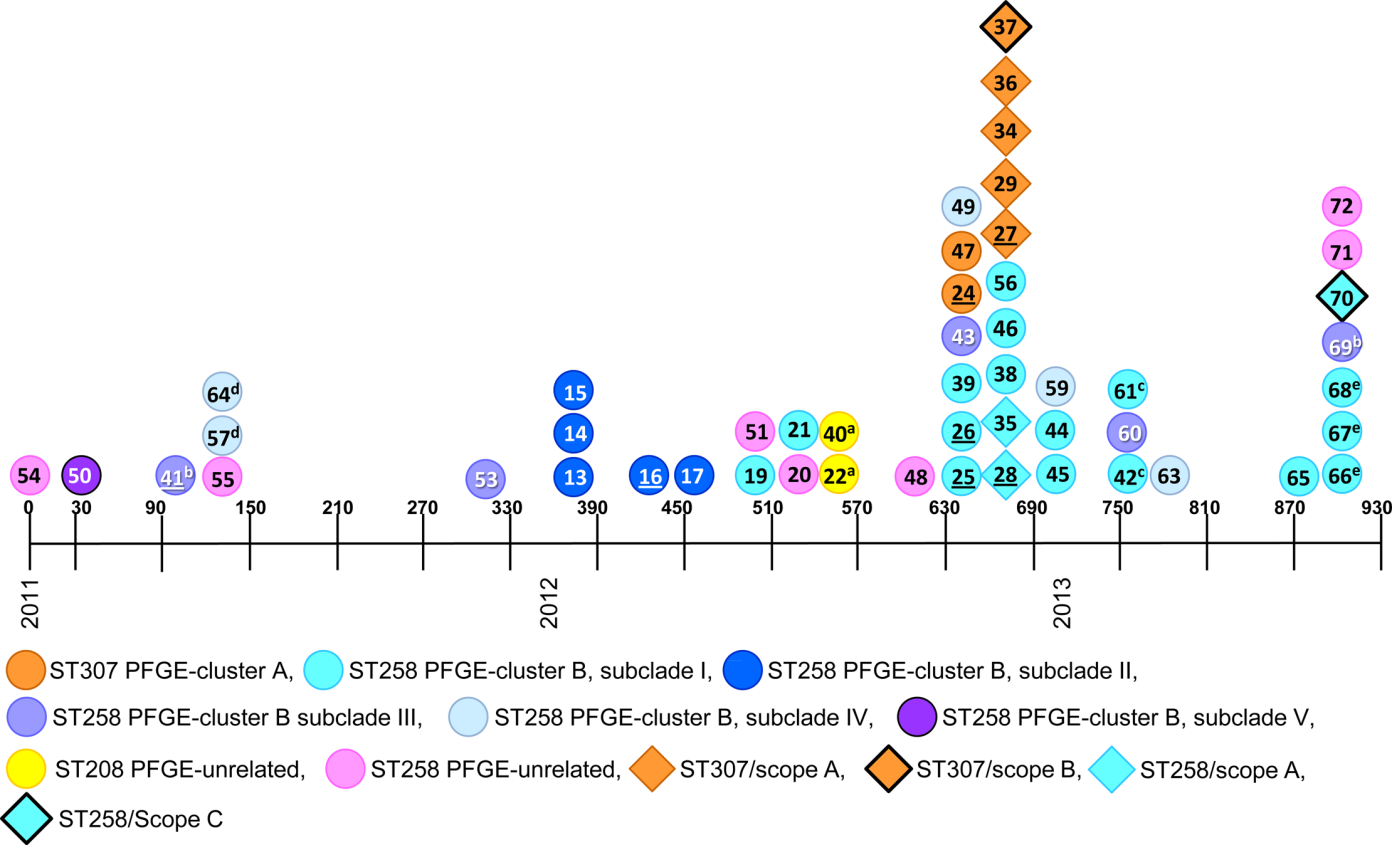
Timeline of 51 Kp isolates collected from January 2011 –July 2013. Dates indicated in days relative to collection of first isolate (0–930); isolate source indicated by shape–circle, patient isolate; diamond, endoscope; genotype indicated by color; underlined IDs depict isolates with WGS; superscripts ^a, b, c, d, e^ represent multiple isolates from the same patient as described in S1 Table.

**Fig 2:**
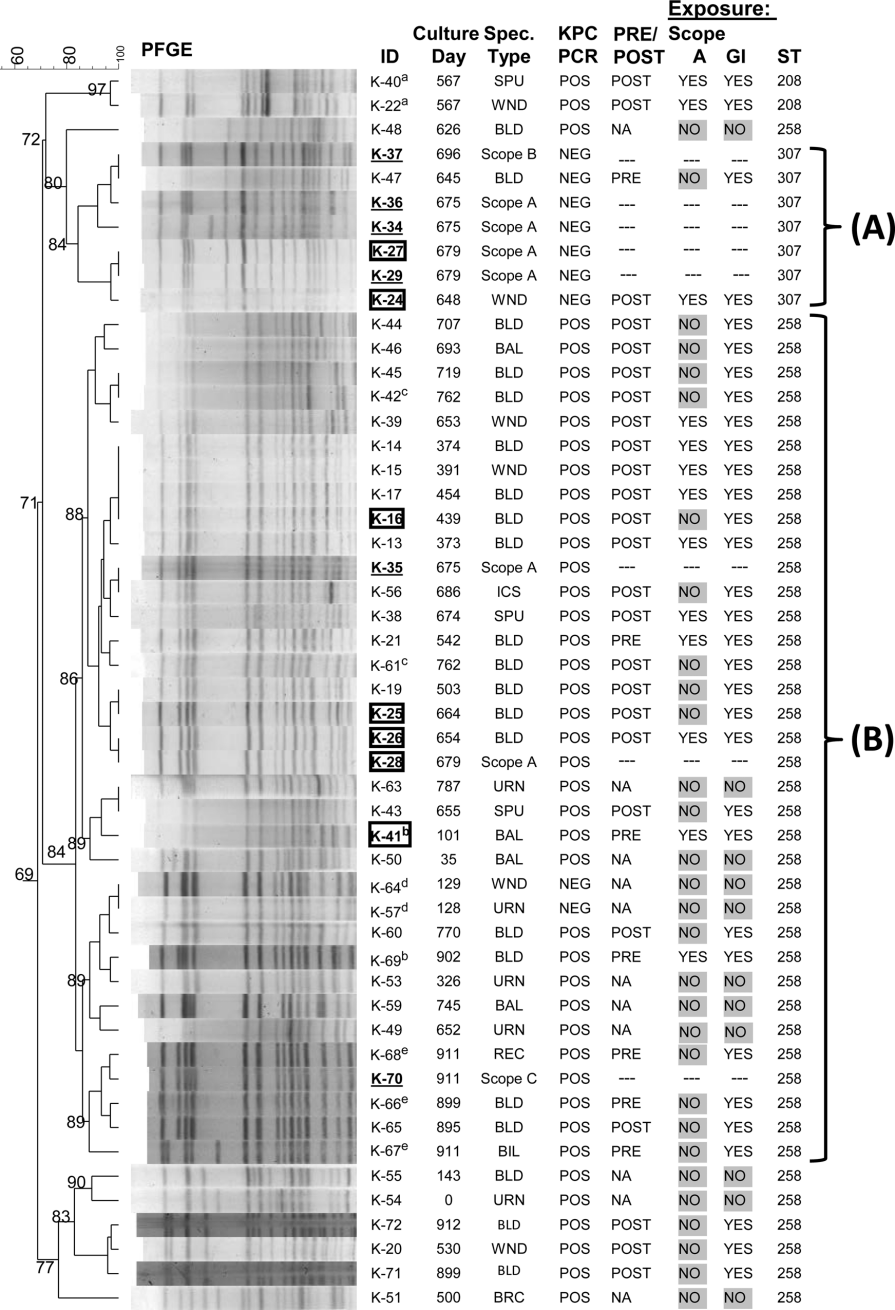
PFGE dendrogram of 51Kp isolates. Cluster A (ST307) and Cluster B (ST258) with ≥84% band similarity with endoscope isolates (ST307, K-27, K-29, K-34, K-36, K-37; ST258, K-28, K-35). Boxed, bold IDs have WGS data. Endoscope IDs bold and underlined, (K-27 and K-28, boxed). Superscripts (a-e) indicate isolates from the same patient. Spec., specimen; GI, gastrointestinal procedure; ST, sequence type; BLD, blood; BAL, bronchoalveolar lavage; ICS, infection control survey; WND, wound; SPU, sputum; URN, urine; REC, rectal swab; BIL, bile; NA, not applicable; PRE, prior to scope exposure; POST, after scope exposure.

A total of 51 Kp isolates collected from January 2011 through July 2013 underwent PFGE genotyping (Figs 1 and 2, S1 Table). There were 42 KPC-Kp isolates– 39 from 34 patients and 3 from 2 endoscopes (Scopes A and C) and 9 non-KPC-producing, ESBL-producing Kp isolates– 4 from 3 patients and 5 from 2 endoscopes (Scopes A and B) (Fig 1 and S1 Table). Of 27 patients with GI procedures, 11 had an ERCP procedure with Scope A. One KPC-Kp culture positive patient unrelated to the outbreak had an ERCP procedure with Scope C. There were 10 patients without history of GI procedures. No patient exposure data were available for Scope B. This study was conducted with the approval of the University of Pittsburgh Institutional Review Board. All patient records were de-identified prior to analysis.

### Antibiotic susceptibility

Susceptibility testing was performed for all Kp isolates and *E*. *coli* transformants (see below) by disc diffusion on Mueller Hinton agar. Zones of inhibition were measured and CLSI breakpoints were used to define susceptibilities (S1 Table).

### PFGE

Pulsed-field gel electrophoresis (PFGE) was performed according to the method of Vimont *et al*. [16]. Band similarities were calculated by the unweighted pair group method with arithmetic mean (UPGMA) with the Dice Coefficient and an 85% cut-off to establish genetic relatedness using BioNumerics v6.6 software (Applied Maths, Austin, TX).

### MLST and *bla*_KPC_ PCR

Genomic DNA (gDNA) from Kp isolates was extracted using a NucliSens easyMag automated instrument (bioMerieux, Durham, NC). MLST of 7 Kp housekeeping genes was performed [17]. Alleles and sequence types (STs) were assigned by querying the PubMLST database (http://bigsdb.web.pasteur.fr/klebsiella/klebsiella.html). Presence of the *bla*_KPC_ gene was determined by PCR using *bla*_KPC_-specific primers [18].

### Genome sequencing and analysis

Whole genome sequencing (WGS) was performed on a subset of 7 Kp isolates using an Ion Torrent PGM^TM^ Sequencer and Torrent Suite software v3.4.2. (Life Technologies, Carlsbad, CA). The genomic DNA was prepared as 200-bp fragment libraries and run on 318 v1 chips according to manufacturer’s protocols (Life Technologies). *De novo* assembly of each of the 7 genomes was performed using Mira 3.9.4 to determine the phylogenetic relationship of the isolates [19]. Repetitive regions and contigs <500 bp were excluded. Annotation of the assembled genomes was performed using the National Center for Biotechnology Information (NCBI) Prokaryotic Genome Annotation Pipeline (PGAP, http://www.ncbi.nlm.nih.gov/genome/annotation_prok/) (May 2013, version 2.0). Single nucleotide polymorphisms (SNPs) were identified by mapping the sequencing reads from all 7 isolates to each genome assembly in a pair-wise fashion using Burrows-Wheeler Aligner and SAM tools [20,21]. SNPs with quality scores <100 or with <75% mapped reads were filtered. SNPs present at more than 5 occurrences over 1,000 bp were excluded to remove SNPs associated with recombination from the analysis. A maximum-likelihood phylogeny of the resulting alignments was generated using RAxML with the default settings [22]. Gene presence/absence was estimated by pairwise comparison of predicted coding sequences from each genome with the sequencing reads. Open reading frames with mapped read coverage ≥15 over the entire length were considered present while those with mapped reads <15 were considered absent. Whole genome shotgun sequences have been deposited to DDBJ/EMBL/GenBank with the accession numbers: K-16, LFNU00000000; K-24, LFNV00000000; K-25, LFNW00000000; K-26, LFNX00000000; K-27, LFNY00000000; K-28, LFNZ00000000; K-41, LFOA00000000.

### PCR confirmation of gene content and pKp28 presence

A total of 9 primer pairs were designed based upon K-41 and K-28 WGS assemblies to confirm and determine gene content among ST258 isolates (S2 Table). Six primer pairs specific to genes flanking or within a 45-kb contiguous region in K-41 were used to demonstrate gene presence or absence by PCR (S1 Fig). Two additional primer sets were used to determine the presence or absence of trehalose synthase or nitrate sensor/regulator genes among ST258 isolates by PCR. Finally, primers specific to a newly described KPC-encoding plasmid, pKp28, were designed to determine plasmid presence or absence among ST258 isolates by PCR. All PCR reactions were performed in 50 ul with 1X AmpliTaq Gold PCR buffer, 2.5mM MgCl_2_, 0.2mM each deoxynucleoside triphosphate, 0.2 uM each primer and 1.5 U AmpliTaq Gold DNA polymerase (Applied Biosystems, Foster City, CA). PCR cycle conditions were: 95°C for 5 min., followed by 35 cycles of 95°C for 1 min, 54°C for 1 min, 72°C for 1.5 min and a final extension at 72°C for 7 min.

### Plasmid analysis

Plasmid diversity among the 35 PFGE Cluster B ST258 KPC-Kp isolates was characterized by S1 nuclease digestion [23,24]. Plasmid profiles (PP) were defined using UPGMA and dice coefficient with 1% optimization and 1.5% band tolerance with BioNumerics v6.6 and lambda ladder PFGE marker for standardization (NEB, Ipswitch, MA). KPC-encoding plasmids from patient isolate K-41 and endoscope isolate K-28 were isolated by transformation of *Escherichia coli* TOP10 [25]. Briefly, electrocompetent cells were transformed with plasmid DNA to obtain transformants with reduced ertapenem susceptibility compared to *E*. *coli* TOP10. Plasmids were purified from these transformants using the Qiagen Maxi Kit according to the manufacturer’s instructions (Qiagen, Valencia, CA). Plasmid pKp28 and pKp41 were sequenced by single molecule, real-time (SMRT) sequencing on a PacBio RS II instrument at the Yale Center for Genome Analysis. *De novo* assembly of a single contig was performed using the hierarchical genome assembly process (HGAP) available in the SMRT Analysis v2.1 software (Pacific Biosciences, Menlo Park, CA) [26]. This molecule was used as reference to reassemble reads and generate a consensus sequence using Quiver v1 of the SMRT Analysis v2.1 software. Plasmid sequences were annotated by NCBI PGAP and submitted to GenBank under accession numbers CP011999 (pKp28), CP012000 (pKp41). A search of the non-redundant BLAST database was performed to identify plasmids with >99% sequence identity to pKp28 and pKp41.

## Results

### PFGE and MLST

PFGE of 43 Kp patient isolates identified 2 clusters with ≥84% band similarity to Kp isolates recovered from 2 ERCP endoscopes (Scopes A and C) and 1 EUS endoscope (Scope B) post high-level disinfection (Fig 2, Clusters A and B). A PFGE cluster containing 2 patient isolates and 5 isolates from 2 endoscopes (Scopes A and B) were ST307 (Fig 2, Cluster A). Isolates from this cluster were ESBL-producing but carbapenemase-susceptible and PCR negative for *bla*_KPC_ (Fig 2 and S1 Table). Patient isolate K-24 was genetically related to a Kp isolate cultured from Scope A (K-27, Fig 2). One isolate (K-47) collected prior to the patient’s GI procedure was indistinguishable from Scope B isolate K-37. Because no information regarding Scope B usage was available, conclusions regarding Kp transmission from Scope B could not be made. However, these data suggest disinfection failure and potential transmission of ESBL-Kp between patients and GI endoscopes.

A second PFGE cluster of 32 isolates from 27 patients and 3 isolates from 2 endoscopes post disinfection were ST258 and defined as the primary outbreak clade (Fig 2, Cluster B). All PFGE Cluster B ST258 isolates were carbapenem -non-susceptible and *bla*_KPC_-positive by PCR except for a pair of isolates from a single patient (K-57 and K-64, Fig 2 and S1 Table). This patient was one of 5 patients who was culture positive for the ST258 outbreak clade and had not undergone any GI procedure. The high similarity by PFGE of Scope A isolates K-28 and K-35 to patient isolates K-26 and K-38 and the fact that these patients had ERCP with Scope A suggested transmission of ST258 KPC-Kp from Scope A. However, 15 patients with ST258 isolates belonging to PFGE Cluster B had no history of Scope A exposure suggesting the outbreak was not limited to a single endoscope and was also due to alternative sources.

Two patients grew ST258 KPC-Kp before ERCP with Scope A. These patients’ isolates (K-21, K-41 and K-69) clustered by PFGE with isolates cultured from Scope A (K-28 and K-35, Fig 2). Patient isolate K-41 was obtained on day 101 of the study, 140 days before the patient’s ERCP procedure with Scope A; isolate K-69 was obtained from the same patient on day 902 of the study, 660 days post Scope A ERCP procedure. Patient isolate K-21 was collected on study day 542, 21 days before ERCP with endoscope A. Notably, this patient had multiple ERCP procedures before becoming KPC-Kp positive suggesting the outbreak was not limited to a single endoscope and was also due to alternative sources including the possibility of additional endoscopes being contaminated with the ST258 outbreak clade. A total of 3 patients who had Scope A ERCP procedures between days 310 and 336 became culture positive for ST258 KPC-Kp between days 373 and 391 and their isolates clustered with Scope A isolates K-28 and K-35 by PFGE (K-13, K-14 and K-15, Figs 1 and 2). These results suggest that Scope A was contaminated before day 373, thus ruling out isolate K-21 and suggesting that isolate K-41 represented the potential index case isolate.

### Comparative whole genome sequence analysis

**Fig 3:**
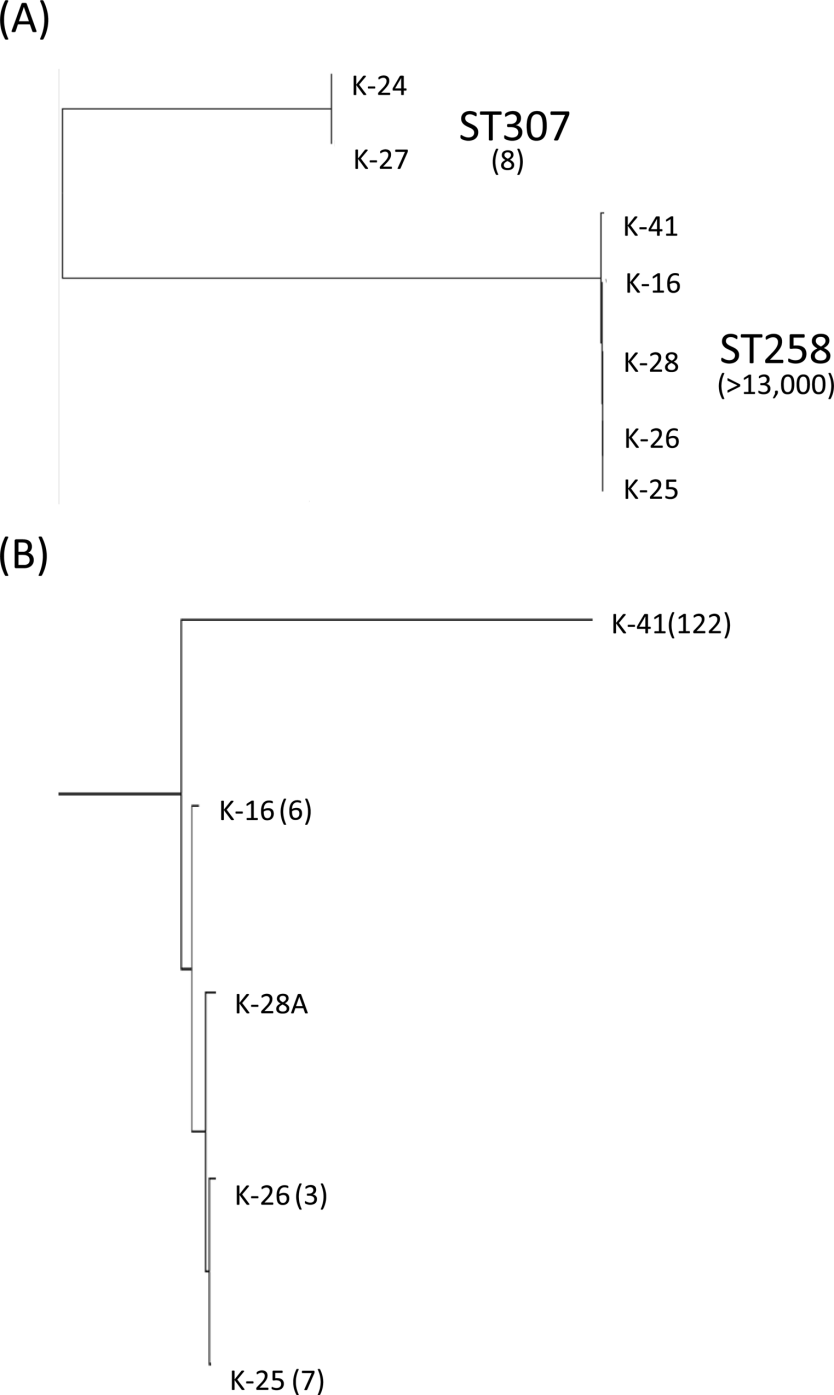
Maximum likelihood trees depicting the phylogenetic relationship of 7 Kp patient and endoscope isolates. (A) 7 isolates from PFGE clusters A and B relative to endoscope isolate K-27 (B) 5 ST258 isolates from PFGE cluster B relative to endoscope isolate K-28. Numbers in parentheses indicate SNP differences.

Whole genome sequence analysis was performed on 5 patient and 2 endoscope isolates selected to represent the diversity across the PFGE dendrogram (Fig 2, strain ID’s boxed and in bold). The phylogeny based on the analysis of SNPs was consistent with the PFGE and MLST data. Two distinct lineages corresponding to ST307 and ST258 were observed on the maximum likelihood tree of all 7 genomes, with >13,000 SNP differences observed between the ST258 and ST307 isolates (Fig 3A, Table 1). A difference of 8 SNPs between patient isolate K-24 and endoscope isolate K-27 was consistent with the PFGE data and supported endoscope-mediated transmission of ST307 ESBL-Kp. Furthermore, fewer than 8 SNP differences were observed among 3 ST258 KPC-Kp patient isolates (K-16, K-25 and K-26) and Scope A isolate K-28 (Fig 3B, Table 1). Taken together, these data suggest that transmission of at least 2 distinct Kp genetic lineages (ST307 and ST258) may have occurred during procedures with ERCP Scope A. The patient with isolate K-25 was not exposed to Scope A; however, this patient did have a GI endoscope procedure in the time period when K-26 was collected, raising the possibility that other endoscopes may have been contaminated with this ST258 clade. More than 120 SNP differences were observed between patient isolate K-41 and the Scope A isolate, K-28 (Fig 3B, Table 1). These data suggest that isolate K-41 was in fact, not the source of the Scope A-associated outbreak.

**Table 1:** SNP differences among 7 *K*. *pneumoniae* isolates.

	ST307		ST258				
	K-27	K-24	K-28	K-26	K-25	K-16	K-41
K-27	0	8	13466	13467	13471	13466	13526
K-24		0	13675	13676	13680	13673	13733
K-28			0	3	7	6	122
K-26				0	4	3	121
K-25					0	7	125
K-16						0	118
K-41							0

In general, the differences in gene content observed among the 7 sequenced isolates were consistent with the SNP phylogeny (Table 2). Over 600 gene differences were observed between ST307 (PFGE Cluster A) and ST258 (PFGE Cluster B) isolates. In contrast, only a few gene differences were observed among endoscope isolates (K-27 and K-28) and patient isolates shown to be phylogenetically related (Table 2). Interestingly, patient isolate K-16, which only differed by 6 SNPs from Scope A isolate K-28, harbored 29 genes that were not present in K-28 (Table 2). Most of these genes are organized into a contiguous 15-kb region predicted to encode a type 1 fimbrial biogenesis operon (S1 Fig). A 30-kb region predicted to encode carbohydrate transport/utilization and fimbrial-like proteins is contiguous with this 15-kb type 1 fimbrial biogenesis operon in isolate K-41 and is absent in patient isolates K-16, K-25 and K-26 and Scope A isolate K-28 (S1 Fig). A BLAST analysis of this 45-kb region from the K-41 chromosome identified genes encoding a transposase, an integrase and a recombinase at the junction of the 35-kb carbohydrate utilization region and the 15-kb type 1 fimbrial operon in recently sequenced ST258 genomes (KPNIH30, KPNIH32, KPR0928) [11]. This finding suggests that differences in gene content among ST258 outbreak isolates arose through several recombination events in this region of the chromosome. In total, 50 of the 70 gene differences between K-41 and the ST258 endoscope-associated isolates were located on this 45-kb region. Taking the SNP and gene differences together further indicates that K-41 was not the index case isolate.

**Table 2:** Gene differences among 7 *K*. *pneumoniae*.

	ST307		ST258				
	K-27	K-24	K-28	K-26	K-25	K-16	K-41
K-27	0	+4	+287	+286	+294	+263	+247
	0	-3	-357	-347	-361	-368	-358
K-24		0	+297	+299	+303	+269	+270
		0	-354	-345	-357	-365	-355
K-28			0	+3	+8	+1	+22
			0	0	-1	-29	-71
K-26				0	+4	+0	+20
				0	-2	-29	-71
K-25					0	+1	+20
					0	-35	-79
K-16						0	+28
						0	-52
K-41							0
							0

In addition, there were ~28 genes present in Scope A isolate K-28 and patient isolates K-16, K-25 and K-26 that were absent in patient isolate K-41 (Table 2). Some of these genes mapped to 2 distinct ~ 6 kb regions on the K-28 chromosome—one predicted to encode a trehalose carbohydrate utilization protein and the other encoding a nitrate/nitrite sensor/regulator.

The differences in gene content identified by WGS among the 35 ST258 outbreak isolates (PFGE cluster B) were explored by PCR. The PCR results confirmed the gene content data obtained by WGS and defined 5 ST258 subclades (Table 3, subclades I-V). Subclade I (19 isolates) is the predominant ST258 genotype associated with the outbreak and includes patient isolates highly related to Scope A (K-28 and K-35) as well as Scope C isolate (K-70, see description in Plasmid diversity) which was not associated with the outbreak. Of the 13 patients with subclade I isolates, 11 patients had GI procedures either with Scope A (n = 3) or with another endoscope (n = 8) prior to becoming ST258 culture positive (S1 Table). Two patients were ST258 culture positive prior to ERCP with either Scope A (K-21) or Scope C (K-66). Subclade I was first observed on study day 503 and persisted until day 911 (Fig 1). Subclade II (5 isolates) was observed among 5 patients who had GI procedures between days 373 and 454 (Fig 1). Four of the 5 patients had ERCP with Scope A (S1 Table) suggesting that Scope A might also have been contaminated with subclade II despite the lack of a corresponding isolate from the endoscope cultures. Subclade III (5 isolates) includes the putative index case isolates (K-41 and K-69), 2 isolates from patients without Scope A exposure but with a history of GI procedures (K-43 and K-60) and 1 additional isolate from a patient without a known epidemiologic link (K-53) (S1 Table). Isolates belonging to subclade III were observed sporadically from day 101 of the study to day 902 (Fig 1). Subclade IV (5 isolates) includes isolates collected from 4 patients with no history of GI procedures and were observed sporadically. Subclade V was represented by a single patient isolate (K-50) with the earliest collection date (day 35) among PFGE cluster B isolates without Scope A or any GI procedure (Fig 1).

**Table 3:** PCR of pKp28 and gene content of 35 ST258 outbreak isolates.

ID	pKp28	carb.util.	fimA	nitrate	trehalose	ST258 subclade
K-19	+	-	-	+	+	I
K-21	+	-	-	+	+	I
K-25	+	-	-	+	+	I
K-26	+	-	-	+	+	I
K-28^a^	+	-	-	+	+	I
K-35^a^	+	-	-	+	+	I
K-38	+	-	-	+	+	I
K-39	+	-	-	+	+	I
K-42^c^	+	-	-	+	+	I
K-44	+	-	-	+	+	I
K-45	+	-	-	+	+	I
K-46	+	-	-	+	+	I
K-56	+	-	-	+	+	I
K-61^c^	+	-	-	+	+	I
K-65	+	-	-	+	+	I
K-66^e^	+	-	-	+	+	I
K-67^e^	+	-	-	+	+	I
K-68^e^	+	-	-	+	+	I
K-70^a^	+	-	-	+	+	I
K-13	-	-	+	+	+	II
K-14	-	-	+	+	+	II
K-15	-	-	+	+	+	II
K-16	-	-	+	+	+	II
K-17	-	-	+	+	+	II
K-41^b^	-	+	+	-	-	III
K-43	-	+	+	-	-	III
K-53	-	+	+	-	-	III
K-60	-	+	+	-	-	III
K-69^b^	-	+	+	-	-	III
K-49	-	+	+	+	-	IV
K-59	-	+	-	+	-	IV
K-57^d^	-	+	+	+	-	IV
K-63	-	+	+	+	-	IV
K-64^d^	-	+	+	+	-	IV
K-50	-	+	+	+	+	V

^a^ endoscope isolates

^b-e^ correspond to multiple isolates from single patients in Figs 1 and 2; carb util., carbohydrate utilization

Taken together with the epidemiologic data, these results suggest that some ST258 subclade I isolates were potentially transmitted by ERCP procedures with Scope A. Furthermore, subclade II may have been associated with Scope A. However, no subclade II isolates were recovered from any endoscopes during culture survey. Although the epidemiology suggests several patient isolates belonging to subclade III could be endoscope associated, the molecular data combined with the sporadic incidence of subclade III isolates indicate otherwise. Subclades IV and V are not associated with ERCP and may represent endemic ST258 strains.

### KPC-encoding plasmids

**Fig 4:**
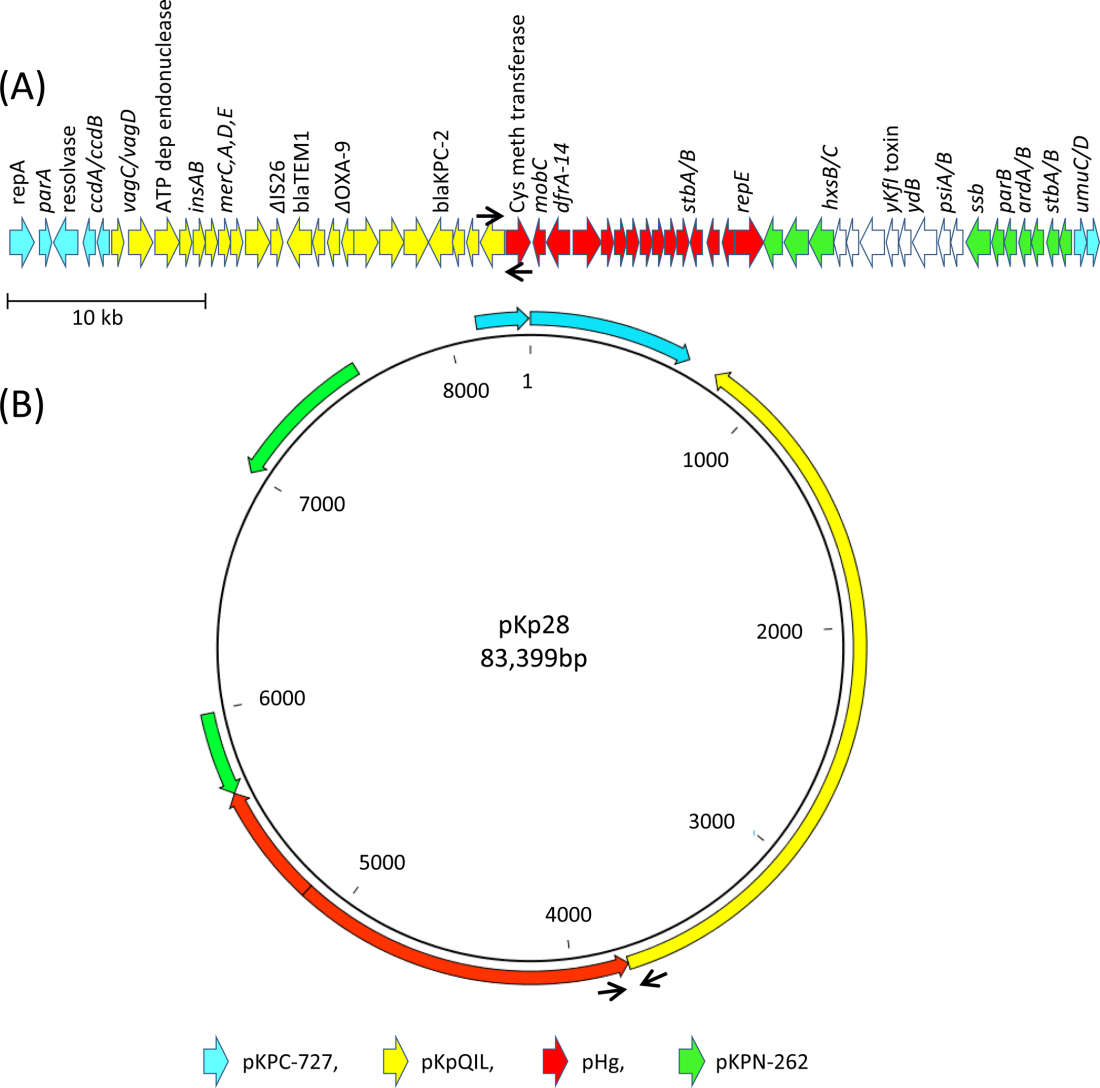
Plasmid pKp28. An 83.4 kb mosaic plasmid with 99% identity to portions of 4 unique plasmids, (blue) pKPC-727; (yellow) pKpQIL; (red) pHg; (green), pKPN-262. (A) Linear depiction of open reading frames (B) circular depiction of pKp28 sequence with >99% nucleotide identity to indicated plasmids. Black arrows indicate PCR primers specific to pKp28.

The subclade structure of the ST258 outbreak is further supported by the identification of a novel KPC-encoding plasmid that was specific to subclade I. SMRT sequencing of the KPC-encoding plasmid from endoscope isolate K-28 identified an 83-kb mosaic plasmid, pKp28 (Fig 4). The *bla*_KPC-2_ gene resides on Tn*4401—*a transposon located on a ~30 kb region showing 99.9% nucleotide identity to pKpQIL, a plasmid commonly associated with ST258 KPC-Kp. An integron-associated *dfrA14* cassette is located on an 18-kb region downstream of *bla*_KPC-2_ with > 99.9% nucleotide identity to plasmid pHg (pKpn2146b), another highly mosaic plasmid identified in KPC-Kp [27]. PCR targeting the pHg/pKpQIL junction in pKp28 was specific to this plasmid (data not shown) and demonstrated that pKp28 was present only in ST258 subclade I isolates (Table 3). These data demonstrate the utility of this unique plasmid for tracking ST258 subclade I. In contrast, sequence analysis of the KPC-encoding plasmid from the putative index isolate, K-41 belonging to subclade III, identified a 113-kb plasmid (pKp41) with 99.9% sequence identity to pKpQIL [28].

### Plasmid diversity

**Fig 5:**
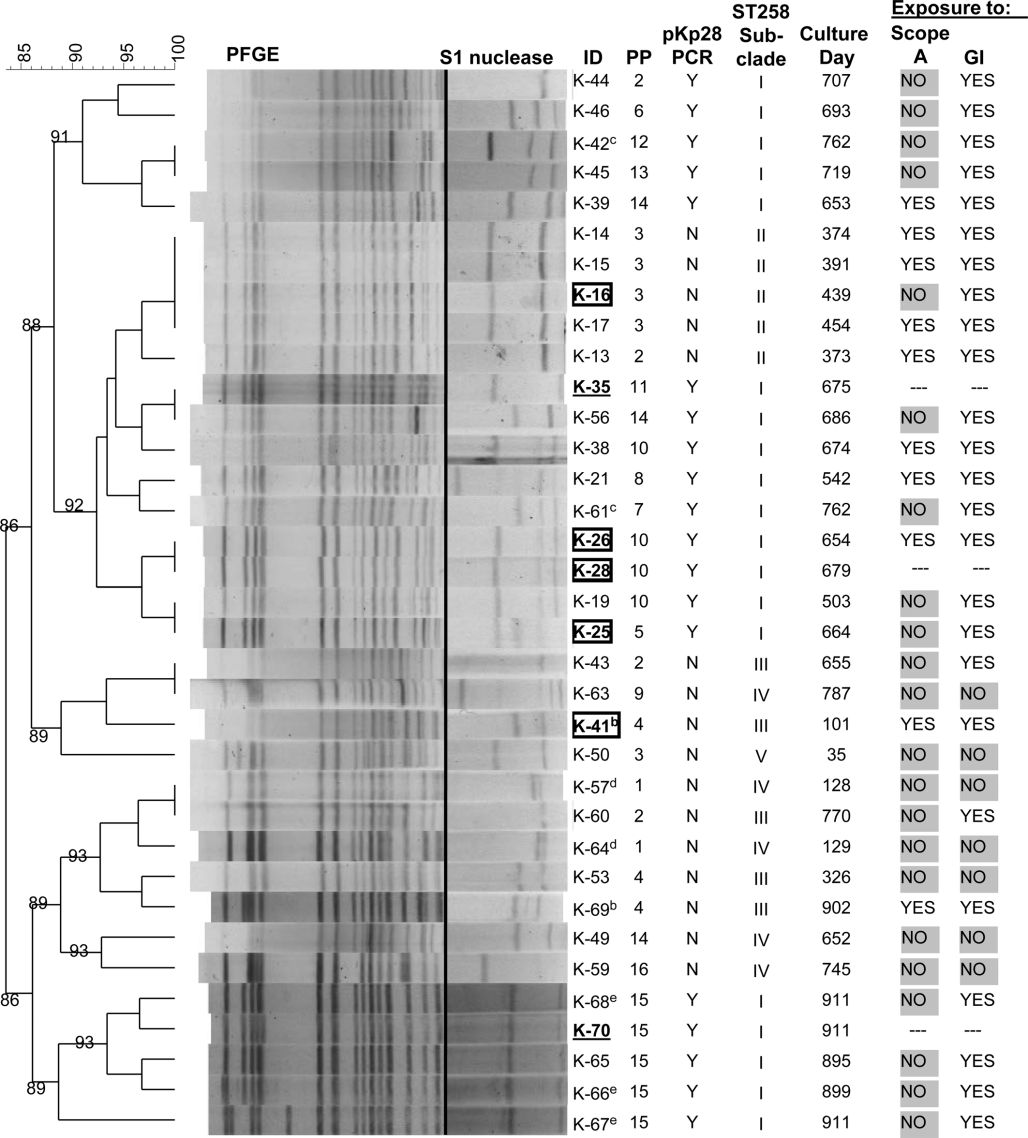
PFGE of 35 Cluster B ST258 isolates with S1 nuclease digests depicting plasmid profiles (PP). Superscripts (b-e) indicate isolates from the same patient. Boxed, bold IDs have WGS data; endoscope IDs are bold and underlined (K-28, also boxed).

At least 16 different plasmid profiles (PP) were observed by S1 nuclease digestion among the 35 ST258 isolates in PFGE cluster B (Fig 5). A few plasmid profiles appeared to correlate with PFGE data and subclade classifications suggesting plasmids may have utility for tracking transmission. For instance, PP15 was unique to a cluster of 5 ST258 subclade I isolates collected over a 17 day period between days 895 and 911 (Fig 5). ST258 subclade I isolates were cultured from different sites from a single patient before (K-66) and on the day of ERCP (K-67 and K-68) with Scope C. Scope C isolate K-70 was recovered after routine high level disinfection. This isolate was highly related to all 3 isolates from the single patient by PFGE and plasmid profiling. These results provide evidence of ST258 KPC-Kp transmission from an infected patient to Scope C.

With some exceptions, other S1 nuclease data suggest that plasmids may have utility in outbreak detection and tracking. For instance, PP10 was observed among 4 closely related ST258 isolates (Fig 5, K-38, K-26, K-28 and K-19) but isolate K-25 which was closely related by WGS to isolates K-28 and K-26 had a different plasmid profile (Fig 5, PP5). PP3 was observed in ST258 subclade II isolates that were indistinguishable by PFGE, but not exclusively. With one exception, isolates from the same patients had the same PP. Subclade I isolate pair K-42 and K-61 differed by both PFGE and plasmid profiling suggesting heterogeneous ST258 subpopulations in a single patient.

Substantial plasmid diversity was observed among ST258 subclade I isolates with 11 different plasmid profiles observed among 19 isolates. These data highlight the dynamic nature of ST258 plasmids and suggest that plasmid exchange occurs frequently even within the same subclade. In some cases, this extreme plasmid diversity may aid discrimination of ST258 isolates where the epidemiology and the genotyping are discordant. For instance, isolates K-43 and K-63 are indistinguishable by PFGE but possess distinct plasmid profiles. This finding combined with the antibiotic susceptibility data support the epidemiology indicating that these isolates are unrelated by time and exposure.

## Discussion

In the current study, a combination of PFGE, MLST, whole genome and plasmid sequencing support endoscope-associated transmission of KPC-Kp and ESBL-Kp. PFGE and MLST identified 2 Kp clusters belonging to ST307 and ST258 and demonstrated genetic relatedness of isolates obtained from endoscopes and patients. These data suggest Kp acquisition after exposure to endoscopes despite high-level disinfection. WGS and phylogenies based upon core SNP analyses were consistent with the PFGE data and further support this conclusion.

The strongest molecular evidence for endoscope-related transmission in this investigation is for patient isolates K-26 and K-38. These patient isolates were indistinguishable or highly related by PFGE, plasmid profiling, SNP (K-26) and gene content to Scope A isolate K-28. Both patients developed ST258 KPC-Kp positive cultures after ERCP procedures with Scope A providing evidence for transmission from Scope A.

Analysis of gene and plasmid content revealed 5 ST258 KPC-Kp subclades. Combined with the epidemiology, these data suggest that the ST258 KPC-Kp outbreak was associated with 2 genetically distinct ST258 subclades (I and II). Most subclade I and II isolates were associated with either Scope A or GI exposures. Subclade IV and V isolates were not associated with any of these exposures indicating ST258 acquisition from another source. Only 2 of the 5 subclade III patient isolates were associated with either Scope A (the putative index case, K-41 and K-69) or another GI endoscope (K-60). However, the finding of >100 SNP differences between K-41 and sequenced subclade I isolates K-25, K-26 and K-28 and subclade II isolate K-16 supports the hypothesis that K-41 was not the index case isolate. Moreover, neither K-41 nor K-60 harbored the pKp28 plasmid characteristic of subclade I and Scope A isolates K-28 and K-35. Thus, the WGS analyses enhanced the epidemiologic investigation.

**Fig 6:**
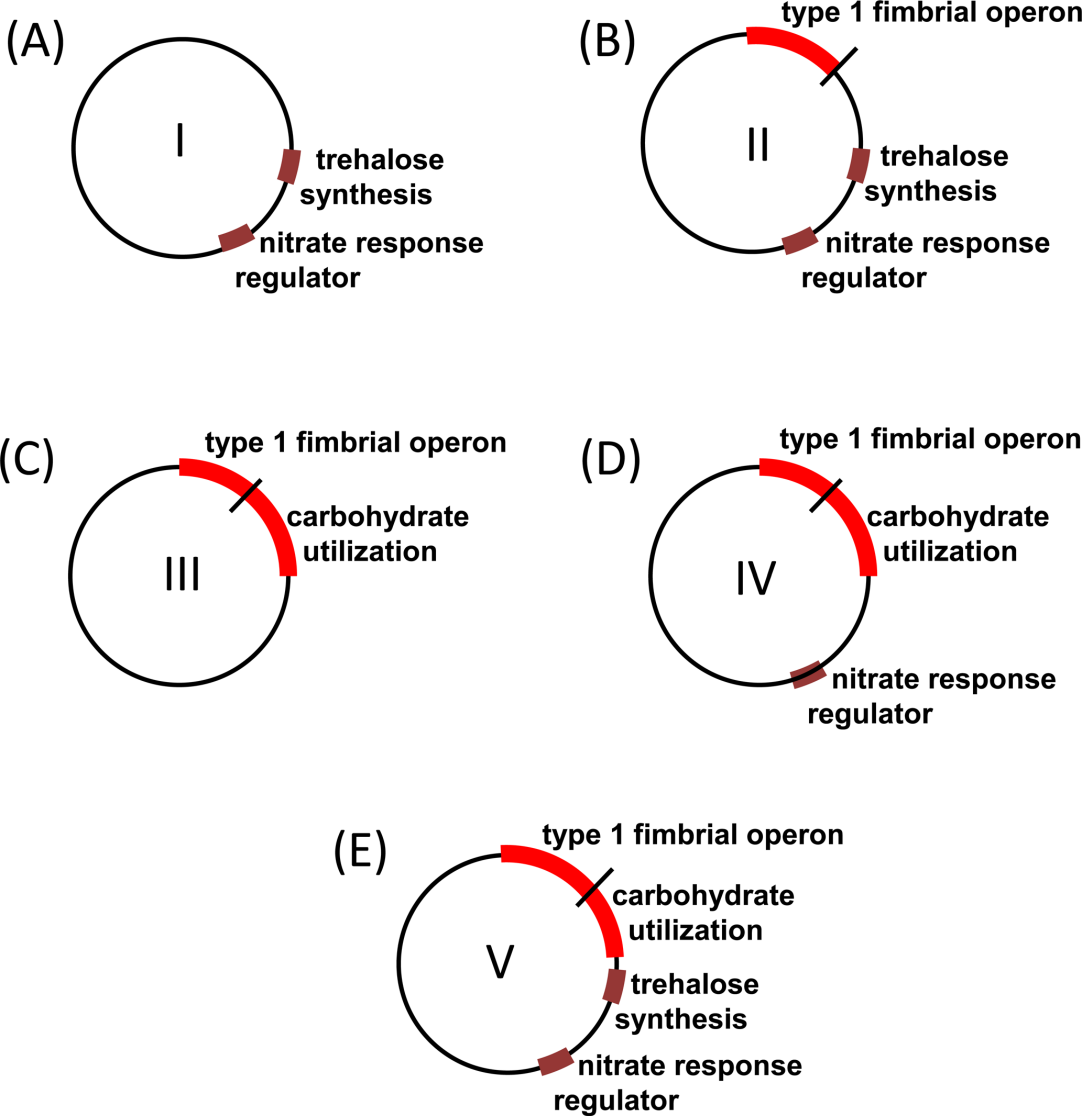
Depiction of gene content defining ST258 subclades in circulation at our institution. (A) subclade I, endoscope-associated; (B) subclade II, endoscope-associated; (C) subclade III, hospital or community-associated; (D) subclade IV, hospital or community -associated, (E) subclade V, recent common ancestor.

The presence of 5 ST258 subclades at our institution could be explained by multiple introductions into the hospital. In fact, multiple ST258 isolates collected during this time period were unrelated by PFGE to cluster B subclades supporting the hypothesis of separate and distinct ST258 introductions. Alternatively, the subclade population structure may be due to ST258 evolution through genetic recombination in the environment of the hospital and/or endoscope. One hypothesis is that the 30-kb contiguous region encoding carbohydrate utilization proteins in K-50 was deleted through genetic recombination giving rise to ST258 subclade II (Fig 6 and S1B Fig). Subclade II may have further evolved through genetic recombination and loss of the 15-kb type 1 fimbrial operon giving rise to subclade I (S1 Fig). WGS supports this hypothesis, as subclade II isolate K-16 is closely related to subclade I isolates K-25, K-26 and K-28 by core SNP analysis.

The substantial plasmid diversity observed among subclade I ST258 outbreak isolates suggests that endoscope-associated plasmid exchange may have occurred between isolates from different ERCP patients. A few stable plasmid profiles were also observed supporting the potential utility of plasmids such as pKp28 for molecular epidemiologic investigations; however, S1 nuclease and PFGE may have limitations in investigations of prolonged outbreaks. Recently, KPC plasmid sequencing revealed genetic signatures enabling investigation of the distribution of KPC-encoding plasmids and their contribution to ST258 KPC-Kp dissemination as well as transmission tracking [11,29]. Thus, whole plasmid sequencing provides high-level resolution necessary to investigate plasmid dynamics and their contribution to clonal emergence and the spread of drug resistance.

The ERCP-mediated outbreak detailed in this investigation is consistent with recent reports citing transmission of carbapenem-resistant *Enterobacteriacae* (CRE) by contaminated duodenoscopes [30,31]. Several recent reviews have cited endoscope-associated outbreaks due to failure of high-level disinfection [32,33]. A review of reprocessing procedures at our institution did not identify any breaches in disinfection procedures. Similarly, in the most recent report of 7 CRE infections and nearly 200 exposures at the University of California, Los Angeles Medical Center, no breach in endoscope reprocessing was identified [34]. The UCLA outbreak prompted the US Food and Drug Administration to issue a safety communication warning healthcare professionals that the complex design of ERCP endoscopes may impede effective disinfection [35].

In summary, this study demonstrates the utility of whole genome and plasmid analysis for identification and molecular characterization of outbreaks due to ST258 KPC-Kp. Phylogeny based upon core SNPs provided objective and improved discrimination compared to PFGE. These data combined with observed differences in gene and plasmid content and the epidemiology provided additional genetic resolution of a highly related ST258 population. Moreover, these data provided insight into the ongoing evolution of ST258 in the hospital setting. Implementation of these types of microbial genomic analyses will aid complex epidemiologic investigations in the future. Finally, this study adds to a growing body of evidence indicating risk of bacterial transmission from ERCP endoscopes.

## Supporting Information

S1 Fig Schematic representation of differences in gene content among sequenced ST258 genomes in this study. K-41 includes a 45 kb carbohydrate utilization genes and type 1 fimbrial operon; K-16 lacks the 30 kb carbohydrate utilization region; K-25, K-26 and **K-28** (endoscope A) lack both carbohydrate utilization genes and type 1 fimbrial operon. Asterisks denote genes targeted for PCR amplification and Table 3 results. Open reading frames are denoted by arrows–pink: (upstream of 45 kb region) gal, transcriptional regulator; blue: (carbohydrate utilization genes) ABC, sugar transporter, CsCR, sucrose operon repressor, fim-like, fimbrial-like protein; orange, (type 1 fimbrial operon) fimA, fimbrial subunit; green: (downstream of 45 kb region) MDR, multidrug resistance protein. Colored triangles depict potential recombination events giving rise to subclade II and subclade I–blue, yields subclade II, orange, yields subclade I.
(PPTX)

S1 Table Epidemiology and susceptibility data from 51 *K*. *pneumoniae* isolates and 2 *Escherichia coli* transformants. (DOCX)

S2 Table Primers used for gene content PCRs. (DOCX)
