# Supplementary material for: Genomic Epidemiology of an Endoscope-Associated Outbreak of Klebsiella pneumoniae Carbapenemase (KPC)-Producing K. pneumoniae
Source: PLoS One. 2015 Dec 4;10(12):e0144310. doi: 10.1371/journal.pone.0144310 (PMC4670079; doi:10.1371/journal.pone.0144310)
Supplement: S1 Fig — K-41 includes a 45 kb carbohydrate utilization genes and type 1 fimbrial operon; K-16 lacks the 30 kb carbohydrate utilization region; K-25, K-26 and K-28 (endoscope A) lack both carbohydrate utilization genes and type 1 fimbrial operon. Asterisks denote genes targeted for PCR amplification and Table 3 results. Open reading frames are denoted by arrows–pink: (upstream of 45 kb region) gal, transcriptional regulator; blue: (carbohydrate utilization genes) ABC, sugar transporter, CsCR, sucrose operon repressor, fim-like, fimbrial-like protein; orange, (type 1 fimbrial operon) fimA, fimbrial subunit; green: (downstream of 45 kb region) MDR, multidrug resistance protein. Colored triangles depict potential recombination events giving rise to subclade II and subclade I–blue, yields subclade II, orange, yields subclade I. (PPTX) [file pone.0144310.s001.pptx]

## Slide 1
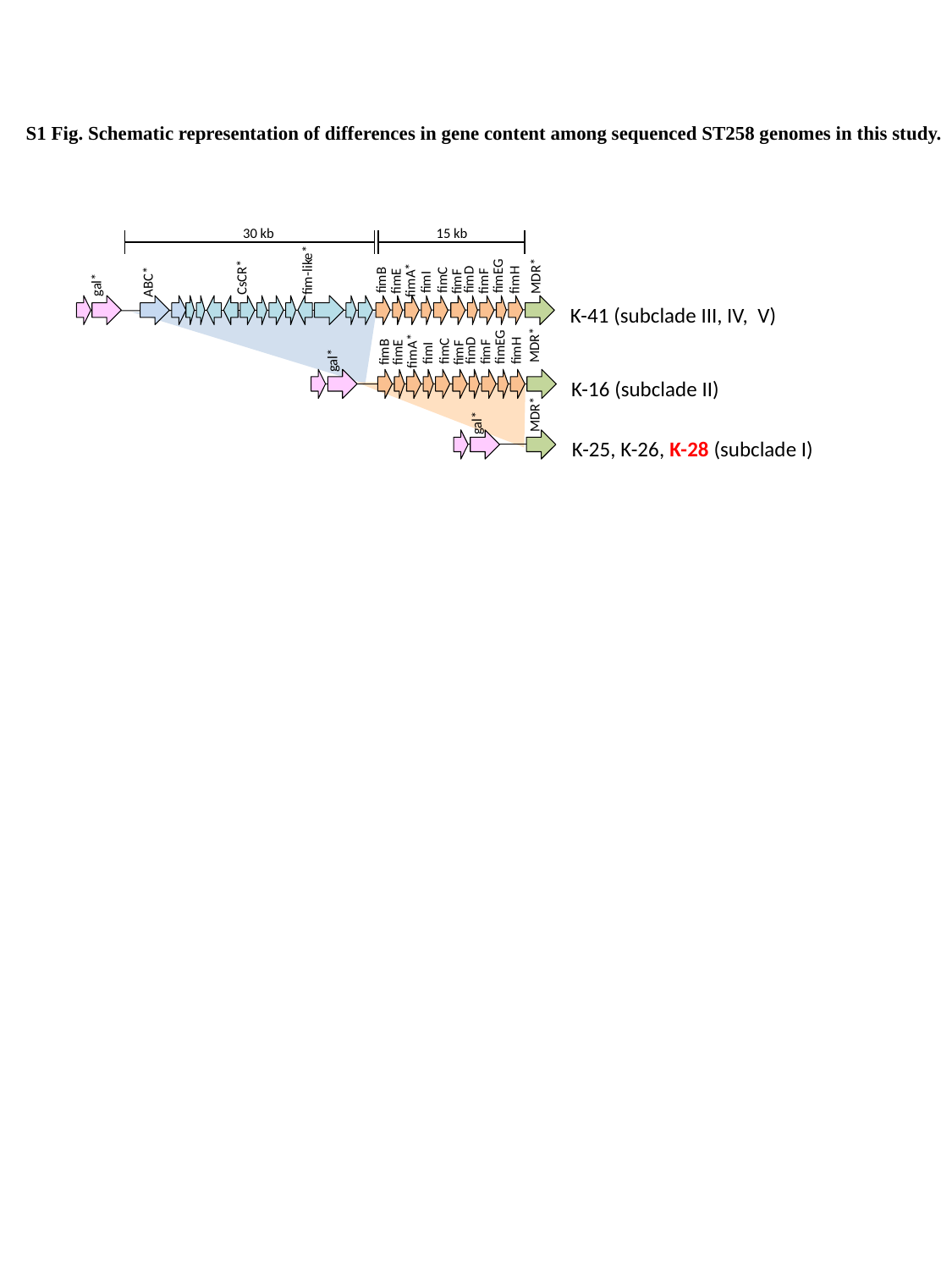

S1 Fig. Schematic representation of differences in gene content among sequenced ST258 genomes in this study.
30 kb
15 kb
fim-like*
MDR*
fimEG
CsCR*
fimH
fimD
fimC
fimA*
fimB
fimF
fimE
fimI
fimF
ABC*
gal*
MDR*
fimEG
fimH
fimD
fimC
fimA*
fimF
fimE
fimB
fimI
fimF
gal*
K-16 (subclade II)
MDR*
gal*
K-25, K-26, K-28 (subclade I)
K-41 (subclade III, IV, V)
