# Supplementary material for: Genomic Epidemiology of an Endoscope-Associated Outbreak of Klebsiella pneumoniae Carbapenemase (KPC)-Producing K. pneumoniae
Source: PLoS One. 2015 Dec 4;10(12):e0144310. doi: 10.1371/journal.pone.0144310 (PMC4670079; doi:10.1371/journal.pone.0144310)
Supplement: S1 Table — (DOCX) [file pone.0144310.s002.docx]

| ID | PFGE Cluster | ST | ST258 Subclade | Source | KPC PCR | Exposure to: | | Culture positive Pre/Post Procedure | Antibiotic susceptibilities (zone of inhibition, mm) | | | | | | | | | | | |
| --- | --- | --- | --- | --- | --- | --- | --- | --- | --- | --- | --- | --- | --- | --- | --- | --- | --- | --- | --- | --- |
|  |  |  |  |  |  | Scope A | GI |  | CIP | CRO | ETP | GM | FEP | IPM | AN | MEM | SXT | TGC | TZP | CL |
|  |  |  |  |  |  |  |  |  |  |  |  |  |  |  |  |  |  |  |  |  |
| K13 | B | 258 | II | Blood | + | Y | Y | Post | 6 | 6 | 11 | 6 | 12 | 15 | 12 | 14 | 6 | 16 | 6 | 12 |
| K14 | B | 258 | II | Blood | + | Y | Y | Post | 6 | 6 | 12 | 7 | 11 | 18 | 11 | 19 | 6 | 20 | 6 | 9 |
| K15 | B | 258 | II | Wound | + | Y | Y | Post | 6 | 6 | 12 | 7 | 11 | 16 | 12 | 15 | 6 | 18 | 6 | 12 |
| K16 | B | 258 | II | Blood | + | N | Y | Post | 6 | 6 | 12 | 7 | 12 | 15 | 12 | 14 | 6 | 18 | 6 | 12 |
| K17 | B | 258 | II | Blood | + | Y | Y | Post | 6 | 6 | 12 | 6 | 11 | 13 | 12 | 12 | 6 | 18 | 7 | 13 |
| K19 | B | 258 | I | Blood | + | N | Y | Post | 6 | 9 | 11 | 18 | 12 | 16 | 11 | 10 | 6 | 20 | 7 | 12 |
| K20 | - | 258 | ND | Wound | + | N | Y | Post | 7 | 10 | 11 | 18 | 12 | 15 | 11 | 9 | 16 | 16 | 8 | 12 |
| K21 | B | 258 | I | Blood | + | Y | Y | Pre | 6 | 6 | 6 | 18 | 8 | 7 | 12 | 6 | 6 | 20 | 6 | 13 |
| K22^a^ | NA | 208 | ND | Wound | + | Y | Y | Post | 35 | 8 | 13 | 7 | 11 | 11 | 22 | 7 | 6 | 23 | 9 | 12 |
| K24 | A | 307 | ND | Wound | - | Y | Y | Post | 6 | 6 | 18 | 10 | 7 | 28 | 22 | 19 | 6 | 22 | 16 | 8 |
| K25 | B | 258 | I | Blood | + | N | Y | Post | 6 | 7 | 7 | 20 | 11 | 13 | 12 | 6 | 6 | 22 | 6 | 12 |
| K26 | B | 258 | I | Blood | + | Y | Y | Post | 6 | 8 | 12 | 23 | 18 | 15 | 12 | 9 | 6 | 20 | 6 | 12 |
| K27 | A | 307 | ND | Scope A | - | NA | NA | NA | 6 | 7 | 18 | 10 | 7 | 28 | 24 | 18 | 6 | 21 | 18 | 13 |
| K28 | B | 258 | I | Scope A | + | NA | NA | NA | 6 | 10 | 12 | 19 | 15 | 20 | 12 | 11 | 6 | 21 | 7 | 12 |
| K29 | A | 307 | ND | Scope A | - | NA | NA | NA | 6 | 6 | 18 | 9 | 6 | 22 | 23 | 19 | 6 | 21 | 17 | 13 |
| K34 | A | 307 | ND | Scope A | - | NA | NA | NA | 6 | 6 | 17 | 10 | 6 | 30 | 26 | 16 | 6 | 24 | 14 | 14 |
| K35 | B | 258 | I | Scope A | + | NA | NA | NA | 6 | 10 | 12 | 20 | 11 | 18 | 13 | 10 | 6 | 21 | 7 | 13 |
| K36 | A | 307 | ND | Scope A | - | NA | NA | NA | 7 | 6 | 17 | 10 | 6 | 29 | 25 | 18 | 6 | 22 | 18 | 13 |
| K37 | A | 307 | ND | Scope B | - | NA | NA | NA | 6 | 6 | 15 | 10 | 6 | 29 | 23 | 17 | 6 | 22 | 16 | 14 |
| K38 | B | 258 | I | sputum | + | Y | Y | Post | 6 | 9 | 11 | 20 | 10 | 17 | 11 | 10 | 6 | 21 | 7 | 13 |
| K39 | B | 258 | I | Wound | + | Y | Y | Post | 6 | 8 | 8 | 22 | 9 | 18 | 13 | 8 | 6 | 20 | 6 | 13 |
| K40^a^ | NA | 208 | ND | sputum | + | Y | Y | Post | 38 | 9 | 12 | 7 | 8 | 12 | 22 | 7 | 6 | 25 | 8 | 14 |
| K41^b^ | B | 258 | III | Bronchioal-veolar lavage | + | Y | Y | Pre | 6 | 6 | 6 | 20 | 6 | 11 | 11 | 6 | 6 | 24 | 6 | 10 |
| K42^c^ | B | 258 | I | Blood | + | N | Y | Post | 6 | 10 | 11 | 19 | 11 | 13 | 13 | 9 | 6 | 19 | 7 | 12 |
| K43 | B | 258 | III | sputum | + | N | Y | Post | 6 | 6 | 6 | 19 | 6 | 11 | 12 | 6 | 8 | 19 | 6 | 13 |
| K44 | B | 258 | I | Blood | + | N | Y | Post | 6 | 6 | 12 | 7 | 11 | 15 | 12 | 15 | 6 | 19 | 8 | 13 |
| K45 | B | 258 | I | Blood | + | N | Y | Post | 6 | 8 | 8 | 20 | 11 | 12 | 12 | 9 | 11 | 20 | 7 | 13 |
| K46 | B | 258 | I | Bronchioal-veolar lavage | + | N | Y | Post | 6 | 7 | 7 | 18 | 10 | 11 | 13 | 9 | 6 | 20 | 7 | 12 |
| K47 | A | 307 | ND | Blood | - | N | Y | Pre | 6 | 6 | 17 | 10 | 6 | 28 | 23 | 23 | 6 | 22 | 19 | 14 |
| K48 | NA | 258 |  | Blood | + | N | N | NA | 6 | 10 | 10 | 20 | 15 | 16 | 13 | 13 | 6 | 21 | 6 | 13 |
| K49 | B | 258 | IV | Urine | + | N | N | NA | 6 | 11 | 9 | 23 | 14 | 13 | 24 | 11 | 6 | 18 | 10 | 13 |
| K50 | B | 258 | V | Bronchioal-veolar lavage | + | N | N | NA | 6 | 10 | 11 | 19 | 16 | 16 | 11 | 14 | 6 | 20 | 7 | 13 |
| K51 | NA | 258 | ND | Bronchoscopy | + | N | N | NA | 6 | 7 | 9 | 8 | 11 | 13 | 12 | 10 | 6 | 22 | 6 | 13 |
| K53 | B | 258 | III | Urine | + | N | N | NA | 6 | 8 | 6 | 21 | 10 | 12 | 14 | 6 | 6 | 17 | 7 | 13 |
| K54 | NA | 258 | ND | Urine | + | N | N | NA | 6 | 11 | 17 | 16 | 18 | 18 | 21 | 18 | 21 | 20 | 14 | 14 |
| K55 | NA | 258 | ND | Blood | + | N | N | NA | 6 | 12 | 15 | 12 | 19 | 18 | 23 | 19 | 7 | 22 | 9 | 8 |
| K56 | B | 258 | I | Infection Control Survey | + | N | Y | Post | 6 | 7 | 8 | 19 | 13 | 15 | 13 | 10 | 6 | 20 | 6 | 13 |
| K57^d^ | B | 258 | IV | Urine | - | N | N | NA | 6 | 11 | 23 | 18 | 17 | 28 | 10 | 29 | 6 | 20 | 17 | 8 |
| K59 | B | 258 | IV | Bronchioal-veolar lavage | + | N | N | NA | 6 | 8 | 13 | 24 | 15 | 17 | 14 | 16 | 6 | 23 | 7 | 12 |
| K60 | B | 258 | III | Blood | + | N | Y | Post | 6 | 9 | 12 | 19 | 15 | 19 | 12 | 16 | 18 | 19 | 7 | 13 |
| K61^c^ | B | 258 | I | Blood | + | N | Y | Post | 6 | 9 | 11 | 19 | 15 | 12 | 13 | 14 | 6 | 19 | 7 | 13 |
| K63 | B | 258 | IV | Urine | + | N | N | NA | 6 | 10 | 12 | 22 | 16 | 16 | 23 | 15 | 6 | 19 | 7 | 13 |
| K64^d^ | B | 258 | IV | Wound | - | N | N | NA | 6 | 10 | 23 | 19 | 16 | 28 | 11 | 28 | 6 | 19 | 17 | 13 |
| K65 | B | 258 | I | Blood | + | N | Y | Post | 6 | 7 | 9 | 21 | 11 | 12 | 12 | 10 | 6 | 20 | 7 | 13 |
| K66^e^ | B | 258 | I | Blood | + | N | Y | Pre | 6 | 7 | 9 | 21 | 12 | 13 | 12 | 12 | 6 | 20 | 6 | 13 |
| K67^e^ | B | 258 | I | Bile | + | N | Y | Pre | 6 | 6 | 6 | 20 | 7 | 6 | 11 | 6 | 6 | 20 | 6 | 12 |
| K68^e^ | B | 258 | I | Rectal | + | N | Y | Pre | 6 | 7 | 6 | 22 | 9 | 9 | 13 | 6 | 6 | 19 | 6 | 13 |
| K69^b^ | B | 258 | III | Blood | + | Y | Y | Pre | 6 | 6 | 6 | 18 | 8 | 11 | 12 | 6 | 6 | 19 | 6 | 12 |
| K70 | B | 258 | I | Scope C | + | NA | NA | NA | 6 | 7 | 6 | 21 | 8 | 6 | 12 | 6 | 6 | 19 | 6 | 13 |
| K71 | NA | 258 | ND | Blood | + | N | Y | Post | 6 | 12 | 13 | 20 | 16 | 14 | 12 | 15 | 18 | 20 | 8 | 13 |
| K72 | NA | 258 | ND | Blood | + | N | Y | Post | 6 | 10 | 10 | 20 | 12 | 13 | 13 | 12 | 18 | 18 | 7 | 14 |
| Ec28^f^ | NA | NA | NA | NA | NA | NA | NA | NA | 50 | 18 | 23 | 32 | 24 | 25 | 23 | 30 | 25 | 15 | 31 | 16 |
| Ec41^g^ | NA | NA | NA | NA | NA | NA | NA | NA | 50 | 18 | 22 | 30 | 22 | 25 | 23 | 30 | 32 | 14 | 30 | 18 |
| TOP10^h^ | NA | NA | NA | NA | NA | NA | NA | NA | 52 | 42 | 42 | 30 | 35 | 48 | 40 | 30 | 34 | 36 | 34 | 17 |
| GI, any gastrointestinal procedure; ^a-e,^indicate multiple isolates from individual patients; ^f-h^ *E. coli* TOP10 transformants; NA, not applicable; ND, not done; CIP, ciprofloxacin; CRO, ceftriaxone; ETP, ertapenem; GM, gentamicin; IM, imipenem; FEP, cefepime; MEM, meropenem; AN, amikacin; SXT, trimethoprim-sulfamethoxazole; TZP, piperacillin-tazobactam; CL, colistin | | | | | | | | | | | | | | | | | | | | |
