# Supplementary material for: Genomic Epidemiology of an Endoscope-Associated Outbreak of Klebsiella pneumoniae Carbapenemase (KPC)-Producing K. pneumoniae
Source: PLoS One. 2015 Dec 4;10(12):e0144310. doi: 10.1371/journal.pone.0144310 (PMC4670079; doi:10.1371/journal.pone.0144310)
Supplement: S2 Table — (DOCX) [file pone.0144310.s003.docx]

| Primer | Sequence 5’-3’ | Size (bp) | GenBank Accession |
| --- | --- | --- | --- |
| gal-F | CTATTAAGGATGTAGCCCGAC | 1003 | LFOA00000000  VZ64_10255 |
| gal-R | TTATCGCCTTCCTGCGG |  |  |
| ABC-F | GCTACGTCGTGCTGATATT | 600 | LFOA00000000  VZ64_10310 |
| ABC-R | CGTTCATCGACCACATAAAC |  |  |
| CsCR-F | TCCGGACGGCATTATCTTTA | 333 | LFOA00000000  VZ64_10345 |
| CscR-R | ATATCGTGGTAATGCTCATCG |  |  |
| fimbrial-like -F | CGAAACATAGCCAACGTAATAGGT | 504 | LFOA00000000  VZ64_10425 |
| fimbrial-like -R | TCGGCGTGGGGTTTTGAG |  |  |
| fimA-F | CCAGCTGGATGATTGCGACAC | 702 | LFOA00000000  VZ64_10490/10495 |
| fimA-R | ACAGCACGTCGGGGTTTT |  |  |
| MDR-F | TTCCCTGTTACGCAAAAAGT | 388 | LFOA00000000  VZ64_10540 |
| MDR-R | GGAGGTGGTCTGCTTAAC |  |  |
| nitrate-F | CTTTACGGTGCAGCTTGATTAC | 888 | LFNZ00000000  VZ63_30885 |
| nitrate-R | GATCATTTTGTTCGGCAGTCC |  |  |
| trehalose synthase-F | GCATTCGCCAAAAGCTACACTAC | 912 | LFNZ00000000  VZ63_13195 |
| trehalose synthase-R | CGCGCTGGTAAACGGACATC |  |  |
| pKp28J2-F | GAGGGCGCAGGGGATTTGAA | 600 | CP011999  ACW84_p00235/p00240 |
| pKp28J2-R | GCGGGGCGGTTGCGATAG |  |  |
